# Fat mass and obesity-associated (*FTO*) rs9939609 polymorphism modifies the relationship between body mass index and affective symptoms through the life course: a prospective birth cohort study

**DOI:** 10.1038/s41398-018-0110-1

**Published:** 2018-03-13

**Authors:** Shinsuke Koike, Marcus Richards, Andrew Wong, Rebecca Hardy

**Affiliations:** 1 MRC Unit for Lifelong Health and Ageing at University College London, 33 Bedford Place, London, WC1B 5JU UK; 2 University of Tokyo Institute for Diversity & Adaptation of Human Mind (UTIDAHM), 3-8-1 Komaba, Meguro-ku, Tokyo, 153-8902 Japan; 3 Center for Evolutionary Cognitive Sciences, Graduate School of Arts and Sciences, The University of Tokyo, 3-8-1 Komaba, Meguro-ku, Tokyo, 153-8902 Japan

## Abstract

Although bi-directional relationships between high body mass index (BMI) and affective symptoms have been found, no study has investigated the relationships across the life course. There has also been little exploration of whether the fat mass and obesity-associated (*FTO*) rs9939609 single-nucleotide polymorphism (SNP) is associated with affective symptoms and/or modifies the relationship between BMI and affective symptoms. In the MRC National Survey of Health and Development (NSHD), 4556 participants had at least one measure of BMI and affective symptoms between ages 11 and 60–64 years. A structural equation modelling framework was used with the BMI trajectory fitted as latent variables representing BMI at 11, and adolescent (11–20 years), early adulthood (20–36 years) and midlife (36–53 years) change in BMI. Higher levels of adolescent emotional problems were associated with greater increases in adult BMI and greater increases in early adulthood BMI were associated with higher subsequent levels of affective symptoms in women. The rs9939609 risk variant (A allele) from 2469 participants with DNA genotyping at age 53 years showed mostly protective effect modification of these relationship. Increases in adolescent and early adulthood BMI were generally not associated with, or were associated with lower levels, of affective symptoms in the *FTO* risk homozygote (AA) group, but positive associations were seen in the TT group. These results suggest bi-directional relationships between higher BMI and affective symptoms across the life course in women, and that the relationship could be ameliorated by rs9939609 risk variant.

## Introduction

Obesity and affective symptoms are common conditions; the life-time prevalence of each is estimated to be greater than 20% in the general population in high-income and middle-income countries^1,2^. Both increase the risk of physical and psychiatric diseases such as cardiovascular diseases, cerebrovascular diseases, diabetes mellitus, and major depressive disorders^1–4^. In addition, among those who develop disease, obesity and depression are associated with poor prognosis and increased mortality^2–4^. Obesity and depression, however, exhibit different patterns across the life course. The prevalence of obesity increases with age until the seventh decade^5^, while the pattern is less clear for affective symptoms. A recent study showed increases during adolescence and decline thereafter^6^, with a gender difference in the prevalence of depression evident from the beginning of adolescence to late adulthood^7^.

A meta-analysis of longitudinal studies of adults showed that overweight and obesity at baseline were associated with an increased risk of depression at follow-up, and that depression at baseline was associated with subsequent higher risk of obesity, but not overweight^8^. Similar bi-directional associations between higher body mass index (BMI) and depressive symptoms have also been found^9–12^, especially in women^9,10,12^. Multiple possible explanations have been proposed for the relationship between higher body weight and depressive symptoms. Shared biological mechanisms such as immunological deficiencies, oxidative stress, neurotransmitter imbalance, and hypothalamic–pituitary–adrenal (HPA) imbalance are likely to play a role^13–15^. This is also likely to be the case for shared developmental and early life environmental risk factors such as low birth weight, perinatal complications, maternal depression, parental socioeconomic status, low childhood cognition, childhood trauma, and chronic stress;^13,14,16^ and shared later life risk symptoms such as insomnia and sleep apnoea^15,17^. Changes in health-related behaviour, such as decreased physical activity, disturbance of eating style, and also decreased self-esteem, are likewise common to both affective symptoms and obesity^13,14^. The relationship between BMI and affective symptoms may change through the life course and be dependent on age at onset of affective symptoms and sex^12^. We previously found that women with adolescent affective symptoms but no adults symptoms had lower BMI in adolescence but greater increases in BMI in adults^12^. Whereas men with adolescent symptoms which continued into adulthood had lower BMI in adolescence and across adulthood. However, no study has investigated the bi-directional relationship between BMI and affective symptoms across the life course.

Genetic studies have repeatedly reported the association between a common variant in the fat mass and obesity-associated (*FTO*) SNP rs9939609 and increased BMI^18–21^. In addition, a meta-analysis of randomized controlled trials suggested that those carrying the rs9939609 risk allele responded equally well to weight loss interventions as those without the risk allele^21^. Although the mechanism and function of *FTO* is not fully understood, possible functional alterations by the gene have been reported in the HPA axis and in the reward system in the brain^22–24^. Disturbances of these systems have also been reported in major depressive disorders, which could lead to several core symptoms such as aberrant emotional and reward responses^4,13^. A prospective cohort study showed an association between another *FTO* SNP rs1421085, which has a strong linkage disequilibrium with the SNP rs9939609, and self-reported depressive symptoms in men, independent of BMI^25^. A clinical case-control study showed an association between the SNP rs9939609 and BMI only for people with major depressive disorder while no association was observed among healthy controls^26^. This is supported by a recent meta-analysis suggesting that affective status modifies the association between the *FTO* genotype and obesity^27^. However, the association may not be seen in Asian populations^28^, and another case-control study including five different ethnicities provided evidence of a protective effect of the rs9939609 obesity-related allele on major depression independent of BMI^29^. There is no longitudinal study which has investigated whether associations between *FTO* and affective symptoms vary over the life course, or whether they may modify the relationship between BMI and affective symptoms.

The Medical Research Council National Survey of Health and Development (NSHD), also known as the 1946 British birth cohort, is one of the longest-running prospective cohort studies, which has repeatedly measured body height and weight throughout life, and obtained affective symptoms over a 40-year period. We previously reported that the relationship between affective symptoms and BMI may differ according to gender and the onset of affective symptoms^12^. We also showed the association between SNP rs9939609 and BMI strengthened during childhood up to the age of 20 and then weakened again during adulthood^19^. The present study aimed first to investigate the possible bi-directional relationship between BMI and affective symptoms through the life course from adolescence to early old age. We hypothesized that, consistent with previous studies, any positive bi-directional relationship between increase of BMI and higher affective symptoms would be seen especially in women. We then investigated whether the *FTO* variant rs9939609 modifies the relationship between BMI and affective symptoms. We hypothesized that the rs9939609 obesity-related A allele would strengthen any positive relationships in an additive model.

## Methods

### Participants

The NSHD is a birth cohort study, initially consisting of a social class-weighted sample of 5362 children drawn from all single births within marriage during 1 week in March 1946 in England, Scotland, and Wales^30,31^. Blood samples for DNA extraction were collected from 2756 members at age 53 years and genotyping were conducted for 2498 participants.

Of 5362 cohort members, 4556 participants had at least one measure of BMI between ages 11 and 53 years (median number of measurements per individual = 5, range = 1–7), and of affective symptoms at ages 13 and 15, 36, 43, 53, and 60–64 years (median = 3, range = 1–5); and are included in the initial analyses investigating the relationship between BMI and affective symptoms. Of these, 2469 participants also had the required information on BMI and affective symptoms and were included in the analysis assessing modification by the *FTO* gene. The participants included in the gene analysis were more likely to be female (*p* < .001), had higher birth weight (*p* = .012), higher overall cognitive score at age 8 years (*p* < .001), and a lower adolescent emotional problem score (*p* < .001) than those excluded.

The study was approved by Multi-Centre Research Ethics Committee for the survey at age 53 and by the Greater Manchester Local Research Ethics Committee and the Scotland Research Ethics Committee for the survey at age 60–64, and written informed consent was given by cohort participants.

### Measures

#### Body mass index

Heights and weights were measured by school doctors at ages 11 and 15 years and by trained nurses using standardized protocols at ages 36, 43, and 53 years, and by self-reports at ages 20 and 26 years. BMI was calculated at each age using the standard weight (kg)/height (m)^2^ formula.

#### Affective symptoms

Adolescent behavioural problems were rated at age 13 and 15 years by teachers using forerunners of the Rutter A scale^32,33^. These ratings have been classified into three behavioural dimensions reflecting emotional (internalizing) problems (e.g., extremely fearful); conduct (externalizing) problems (e.g., a quarrelsome and aggressive child); and lack of self-control (e.g., a poor worker or lazy)^34^. An emotional problem score was calculated from the standardized sum of the relevant factor scores at both ages. Affective symptoms at age 36 years were assessed using a short version of the Present State Examination (PSE), a clinically validated interview administrated by trained nurses^35^. We used the index of definition scale score (range 1―7)^35^. At age 43 years, the Psychiatric Symptom Frequency (PSF) scale was obtained with an 18-item self-reported questionnaire (range 0–90)^36^. At ages 53 and 60–64 years, the 28-item version of the General Health Questionnaire (GHQ-28) was adopted for self-reported depression and anxiety^37^. Each item was scored using a 4-level Likert scale, and was recoded into 0-0-1-1. Therefore, the score ranged from 0 to 28. Given the variation in assessment of affective symptoms, we used the continuous variables in analyses.

#### Genotyping

DNA was extracted and purified from whole blood using the Puregene DNA Isolation Kit (Flowgen, Leicestershire, UK), according to the manufacturer’s protocol. The rs9939609 and rs1421085 SNPs were typed by Source Bioscience PLC using the Applied Biosystems (Foster City, CA, USA) SNPlex technology which is based on an Oligonucleotide Ligation Assay combined with multiplex PCR amplification and capillary electrophoresis. Genotyping was performed using an ABI 3730xl DNA Analyser and ABI GeneMapper v4.0 software. The integrity of the genotyping was checked by genotypeing frequency, concordance between duplicates and Hardy–Weinberg equilibrium (HWE). The call rate was 99.5% with >95% concordance between duplicate samples and there was no evidence of deviation from HWE (*p* > .05). Since the SNP rs1421085 and rs9939609 were in the strong linkage disequilibrium (*r*^2^ = 0.89), our primary analysis focused on the rs9939609 polymorphism.

### Statistical analysis

**Fig. 1:**
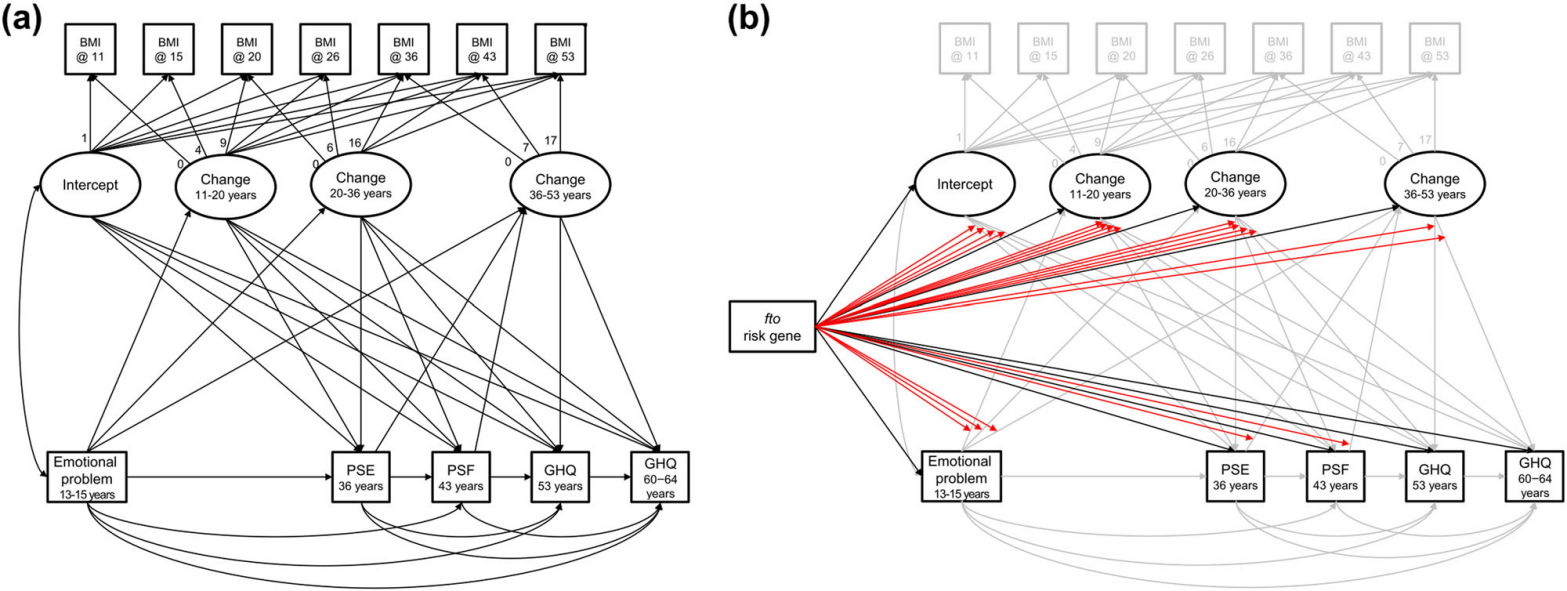
Structural equation modelling for the relationship between BMI and affective symptoms. **a** The correlation and causal relationship between BMI and affective symptoms were set in accordance with time series. **b** In the genetic modification model, the genetic association with four latent BMI variables and five observational depression variables, and 20 gene interactions to the relationships between BMI and affective symptoms, were added

Our previous studies have indicated sex differences in the trajectory of both BMI^19,38^ and affective symptoms^39^, and the relationship between them^12,40^. Therefore, the analysis was stratified by sex if a following structural equation modelling (SEM) showed significant sex differences in BMI and affective symptoms, and interactions in the correlations between BMI and affective symptoms. First, a linear growth model was fitted to the BMI trajectory using four latent variables (intercept at age 11 years and changes from age 11–20 (adolescent), 20–36 (early adulthood), and 36–53 (midlife) years) within a SEM framework. Then we added the adolescent emotional problem score, allowing it to be correlated with the intercept of the BMI trajectory and with all latent variables representing BMI change; and the PSE score at 36 and PSF score at 43, allowing each to be correlated with BMI change from 36–53 (Fig. 1a). Each BMI variable was also related to all subsequent affective symptoms. Relationships between affective symptoms at all ages were assumed. Therefore, each relationship between BMI and affective symptom was tested considering previous measurement results of BMI and affective symptoms. Although BMI at age 60–64 years were available, previous analyses showed a reduction in the rate of BMI increase between age 53 and 60–64 years compared to between age 36 and 53 years^38^. Therefore, to maintain a good statistical fit in a linear growth model with four latent variables, BMI at age 60–64 years was not included in the model. The estimation of the model was conducted using robust maximum likelihood estimation, and missing values were handled using full information maximum likelihood (FIML).

To test genetic modification of the associations between BMI and affective symptoms, the same model was fitted to the subsample with available genetic data and the *FTO* genotype was related to each of the four latent variables for BMI and the five observed affective symptoms variables, and the 20 gene by phenotype interactions were added (Fig. 1b). The rs9939609 A allele was considered a high risk for higher BMI and affective symptoms in an additive genetic model. Where there was evidence of effect modification, multiple-group analysis fitting the initial SEM model (Fig. 1a) was conducted to estimate the correlations between BMI and affective symptoms within each genotype group.

Statistical significance was set at *p* < .05. Good model fit was set at comparative fit index (CFI) >.90 and root mean square error of approximation (RMSEA) <.10. SEM was performed using the “lavaan” package version 0.5-16 in R version 3.1.1^41,42^.

## Results

Male participants had higher BMI compared to female at ages 11–43 years, and fewer affective symptoms at all ages (Table 1). Male participants were also more likely to have TA and less likely to have TT genotypes than female.

**Table 1:** Characteristics of the analytic sample from the MRC NSHD

	Male		Female		*p-*value^b^
	*n*	Mean (SD)	*n*	Mean (SD)	
BMI (kg/m^2^)					
11 years	2050	17.3 (2.1)	1887	17.5 (2.6)	0.006
15 years	1881	19.6 (2.4)	1700	20.6 (3.0)	<.001
20 years	1829	22.6 (2.5)	1735	21.8 (2.9)	<.001
26 years	1822	23.4 (2.8)	1782	22.4 (3.2)	<.001
36 years	1632	24.8 (3.2)	1648	23.6 (4.1)	<.001
43 years	1617	25.7 (3.5)	1608	25.2 (4.8)	<.001
53 years	1452	27.4 (4.0)	1496	27.4 (5.5)	0.81
Depressive symptoms					
Adolescent emotional problems^a^	2044	−0.09 (0.96)	1883	0.10 (1.03)	<.001
PSE score at age 36 years	1640	1.7 (1.1)	1653	2.2 (1.3)	<.001
PSF score at age 43 years	1600	9.6 (10.0)	1585	12.6 (11.8)	<.001
GHQ score at age 53 years	1423	1.9 (3.9)	1479	3.2 (4.9)	<.001
GHQ score at age 60–64 years	1048	1.7 (3.1)	1137	2.8 (4.9)	<.001
*FTO* rs9939609 genotype, TT/TA/AA [%]	1238	414/619/205 [33.4/50.0/16.6]	1233	460/534/239 [37.3/43.3/19.4]	.004^c^

*BMI* body mass index, *PSE* a short version of the Present State Examination, *PSF* the Psychiatric Symptom Frequency scale, *GHQ* the 28-item version of the General Health Questionnaire, *FTO* fat mass and obesity-associated gene

^a^ Z scores from teacher-rated measures at age 13 and 15 years

^b^ Gender differences were tested using *t*-tests, except for the genotype where chi-square tests were used

^c^ Residual analysis showed that male participants were less likely to have TT and more TA genotypes than female participants

### Association between BMI and affective symptoms through the life course

The initial SEM model for all participants had a good fit (*n* = 4556, CFI = .919, RMSEA = .049). The model had the main effects of sex on BMI and affective symptoms, as well as sex interactions on the relationship between BMI and affective symptoms (Supplementary Fig. s1). Thus, further analyses were stratified by sex.

**Fig. 2:**
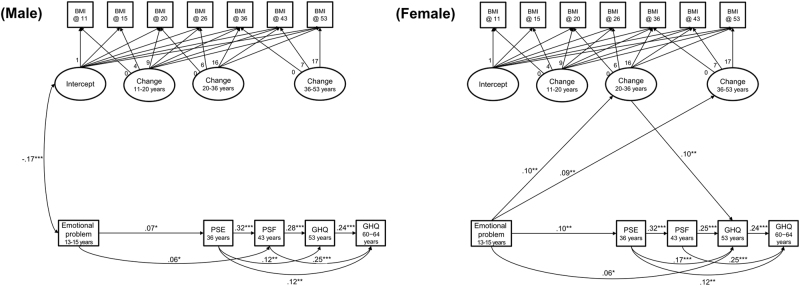
The relationship between BMI and affective symptoms through the life course. Analysis was stratified by sex. Only significant relationships are displayed (*p < .05, **p < .01, ***p < .001)

The SEM model specified in Fig. 1a had good fit in males (*n* = 2357, CFI = .969, RMSEA = .051) and females (*n* = 2199, CFI = .920, RMSEA = .085). Figure 2 displays the significant correlations. In males, there was a negative correlation between BMI at age 11 years and adolescent emotional problems, but no other associations between BMI and affective symptoms were significant at the 5% level (Fig. 2a). In females, adolescent emotional symptoms were positively associated with increases in BMI from ages 20 to 36, and from ages 36 to 53 (Fig. 2b). In turn, increases in BMI from ages 20 to 36 were positively associated with GHQ depression and anxiety scores at age 53.

### Genetic modification of the relationship between BMI and affective symptoms

**Fig. 3:**
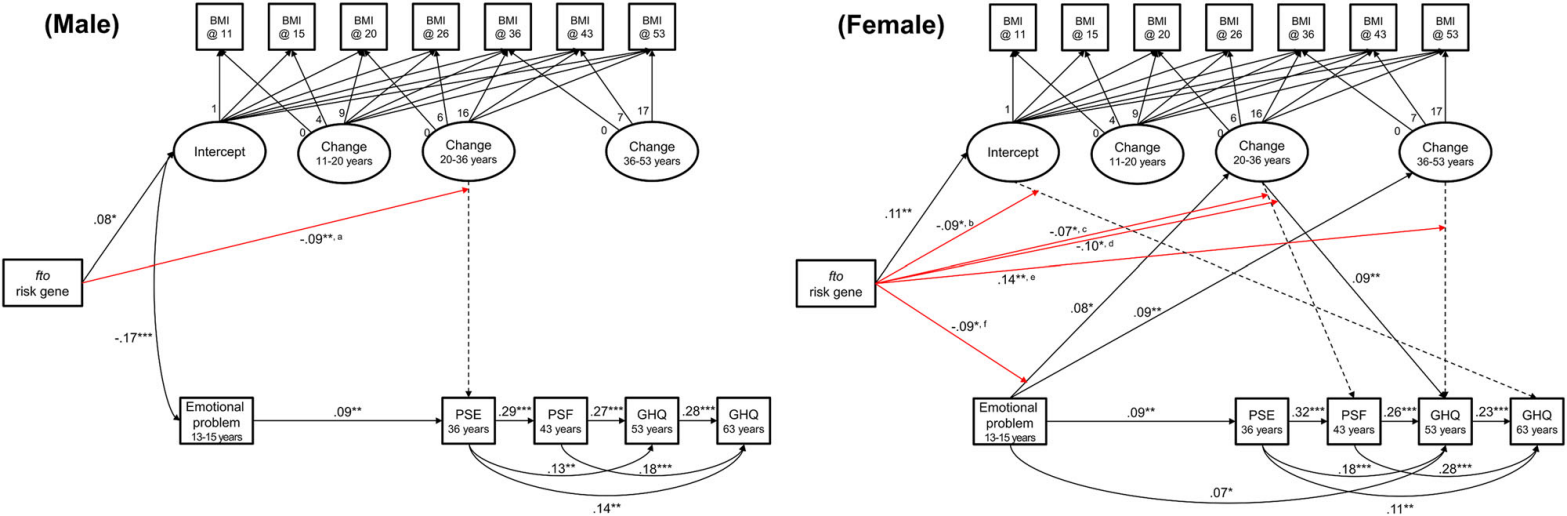
Gene association with BMI and affective symptoms. Analysis was stratified by sex. Only significant relationships are displayed (*p < .05, **p < .01, ***p < .001). Significant gene interaction was shown in red, and a letter on the line indicates that further analysis of the relationship is presented in Fig. 4. The dashed lines represent a non-significant relationship which are modified by the gene

The genetic modification model specified in Fig. 1b had good fit in males (*n* = 1236, CFI = .939, RMSEA = .043) and females (*n* = 1233, CFI = .934, RMSEA = .045). Figure 3 displays the significant correlations. In both sexes, the rs9939609 A allele was associated with higher BMI at age 11 years, but not with subsequent rates of change (Fig. 3).

**Fig. 4:**
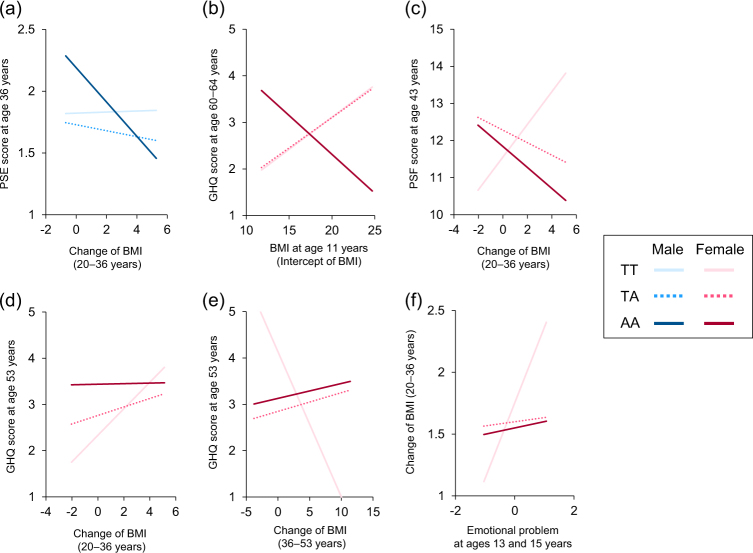
Association between BMI and affective symptoms by *FTO* rs9939609 genotype. The results of multiple-group analysis for the initial SEM model (Fig. 1a) by *FTO* rs9939609 genotype are illustrated using equal loadings, means, residuals, and intercepts for the non-significant effect of genetic modification. In Fig. 4b, the line of TT had an almost overlap with that of TA

There were significant genetic interactions for both sexes; one in men and five in women (Fig. 3). In men, the negative correlation between greater BMI change between ages 20–36 and lower subsequent affective symptoms (at age 36) was stronger in the AA group than in the TA and TT groups (Fig. 4a). Among women, there was a positive relationship between BMI at 11 and GHQ score at 60–64 in the TT and TA groups but a negative association in the AA group (Fig. 4b). There was a strong negative association between BMI change between 20–36 and affective symptoms (at age 43) in the AA group but a positive association in the TT group (Fig. 4c). A similar positive association was observed between change in BMI over the same period and affective symptoms at 53 for the TT group, but there were null associations in the TA and AA groups (Fig. 4d). Similarly, greater changes in midlife BMI were associated with lower levels of symptoms at 53 in the TT group only (Fig. 4e). Finally, there was one suggestion of effect modification of an association from affective symptoms to BMI. The TT group showed a positive association between emotional problems in adolescence and greater change in BMI from 20–36 which was not observed in other genotype groups (Fig. 4f). The coefficients for the saturated models including all interactions with the *FTO* genotype are shown in Supplementary Tables S1–2.

The results for the SNP rs1421085 are provided in Supplementary Materials and Fig. S2. The results are largely similar except for the gene modification of the association between BMI change between 20–36 and affective symptoms at age 36 but not at age 43 in women.

## Discussion

In a representative general population prospective cohort study we found that adolescent but not adult emotional problems were associated with BMI and that the relationships were different for men and women. A higher emotional problem score was associated with lower BMI at age 11 in men, while a higher emotional score was associated with greater midlife increases in BMI in women. In turn, greater midlife increases in BMI were associated with higher levels of affective symptoms at age 53 years in women. There was evidence that *FTO* rs9939609 modified these associations, particularly in women. In women greater increases in early adult BMI tended to be associated with lower levels of affective symptoms in the TT group (those with lower risk of overweight), whereas there were null or positive associations in the TA and AA groups, contrary to our hypothesis.

Strengths of this study include the use of a national population-based sample with repeated objective measurement of body height and weight, and well-validated assessment of affective symptoms through the life course. This means that we were able to assess the bi-directional relationship between BMI and affective symptoms across a 50-year period. To our knowledge, this is also the first study to report evidence of an effect modification of these associations by the *FTO* risk gene. The use of SEM and FIML meant that we were able to include a large sample size in the initial analysis investigating relationships between BMI and affective symptoms.

Several limitations should be considered. First, although the analytic approach used maximized the sample size, the disproportionate loss to follow-up of those of lower birthweight and from a less socially advantaged background may have affected the findings. However, in this cohort, higher birthweight is only weakly correlated with higher BMI in adulthood^43^ and lower birth weight is associated with adolescence but not adulthood affective symptoms^44^ and is therefore unlikely to have influences the findings to a great extent^45^. BMI is socially patterned according to both childhood and adult social class. Further, socioeconomic position and socially patterned health behaviours such as smoking and physical activity have been found to modify the association of genes with BMI, with the influence of genes being stronger in those from less advantaged groups and those with unhealthy behaviours^46,47^. Hence, the differential loss of those from a more disadvantaged background who are likely to have higher BMI and more unhealthy behaviours may have led to an underestimate of associations in our study. More importantly, DNA was collected for the first time at age 53 years when 2989 participants were interviewed and hence the sample for the analyses including the *FTO* gene was reduced. In particular, the participants included in the genotype analysis had less adolescent emotional problems than those excluded, which may have resulted in a loss of statistical power in analyses. The higher cognitive capacity in those maintained in the analytic sample may also decrease the power to detect the relationship of interest since low childhood cognition is a risk factor for affective symptoms as well as increased body weight. In addition, some of those excluded have died from obesity-related diseases such as coronary heart disease. Therefore, we cannot rule out the possibility that the estimated relationships may be biased, but the missing data may mean that associations are underestimated. However, at age 53, the sample remained broadly representative of the general source population in Britain^48^ and there was little difference between the estimated coefficients from the initial SEM model on the maximum sample and the model on the sample with genotype information. Second, since the effect of the rs9939609 polymorphism on major depressive disorder has been found to vary by ethnicity^28,29^, the results may be different in samples containing other ethnic groups. However, the results from a large sample study including five different ethnicities were similar to our findings^28,29^. Third, although the scales used to measure affective symptoms at each age were well validated, the difference in scales used could influence the relative strengths of the relationships observed. Finally, we acknowledge that there may be alternative models to the theoretical model that we tested. For example, it may be that concurrent changes in symptoms, rather than prior symptoms, are associated with changes in BMI.

The present study found bi-directional associations between BMI and affective symptoms in females but not males. This reinforced the findings of large cross-sectional studies showing the relationship to be stronger in women than in men^49–51^. Previous longitudinal studies have shown that depressive episodes in adolescent girls were associated with obesity in adulthood^10,52^. One reason for these relationships being observed only in women may be because depressive symptoms are more associated with decreased physical activity and eating style disturbance in women than in men^52^, which are also risk factors for obesity. Second, body image dissatisfaction, which is associated with affective symptoms, is more common in women than men^53–55^. A longitudinal study of the effect of weight-loss surgery showed that weight loss improved affective symptoms, in young women particularly^56^. Third, the experience of pregnancy and childbirth may influence this association. Adolescent depressive symptoms raise the risk for postpartum depression^57^, and are associated with weight retention after childbirth;^58,59^ and higher parity was associated with higher BMI at age 53 years in NSHD^60^. Finally, emotional stress responses and related hormone secretion in the HPA axis are greater in females^7,53,61^, which can influence weight increase. In addition, gonadal hormones influence both mood and body weight^7,53^.

The present study showed that the *FTO* rs9939609 risk variant exerted mostly protective effects in relation to affective symptoms for those with greater increases in BMI. Although the mechanism underlying these findings remains unknown, and these findings need to be replicated in view of the multiple interactions tested, one possible explanation may be that adult BMI is partially determined by differences in physical development in adolescence and young adulthood leading to differences in body composition. The *FTO* gene is associated with skeletal muscle as well as subcutaneous adipose tissue^62^, and *FTO* deletion leads to a severe loss of muscle mass in humans and mice^18^. *FTO* mRNA expression is abundant in skeletal muscle in young adults compared to older people^62^. In addition, *FTO* polymorphisms can modify the secretion of growth hormone^63^ and testosterone^64^, which are essential hormones for physical development and muscle growth. Thus, the *FTO* genotype-related increase in BMI in adolescence and young adulthood would be essential for physical development, which could lead to or result from better psychological health. Second, the rs9939609 A allele is protective of child emotional problems and attention deficits and hyperactivity disorder symptoms^65^, which is thought to be due to an altered reward response in dopaminergic neuron in the prefrontal cortex^66^. Conversely, the mouse knock-out *FTO* gene within the midbrain dopaminergic system showed hyperactivity and aberrant sensitivity to dopamine stimulants^23^. Modification of the dopaminergic response in the prefrontal cortex by rs9939609 A carriers may be protective against the detrimental effect of increases in BMI on emergence of affective symptoms. Third, changes in BMI and affective symptoms for individuals with the protective allele could be more influenced by environmental factors such as health behaviours, while changes in those with the risk allele may be more influenced by genetic effect on their BMI. Thus unhealthy behaviours related to affective symptoms such as emotional eating and physical inactivity may be more influential in determining BMI among those with the protective allele. Previous studies have demonstrated modification of the association between genotype and BMI by health-related behaviours such as diet and physical activity^20^. There may be therefore more complex interactions between BMI change and health-related behaviours related to the risk gene and affective symptoms.

Our results showed that BMI and affective symptoms exhibit bi-directional positive relationships across the life course in women only. The *FTO* rs9939609 risk variant may be protective against affective symptoms in men, and particularly in women, who experience high increases BMI in adolescent and early adulthood. We have carried out multiple tests for effect modification of associations between BMI and affective symptoms by the *FTO* gene with inconsistent findings between men and women and between ages. The results should therefore be interpreted with some caution as they could be chance findings and require replication in other cohorts. The long-term effect might be caused by physical and psychological growth, along with the potential biological backgrounds such as hormonal and dopaminergic neuron system, rapidly occur during adolescence. Studies combining epidemiological investigation with biological measurement including gene polymorphism, muscle composition, and hormone concentration may be able to disentangle the complex relationship between BMI and affective symptoms and the *FTO* genotype according to age and gender.

## Electronic supplementary material

Supplementary materials

Supplementary figure S1

Supplementary figure S2

Supplementary Tables
