# Supplementary material for: Fat mass and obesity-associated (FTO) rs9939609 polymorphism modifies the relationship between body mass index and affective symptoms through the life course: a prospective birth cohort study
Source: Transl Psychiatry. 2018 Mar 13;8:62. doi: 10.1038/s41398-018-0110-1 (PMC5847566; doi:10.1038/s41398-018-0110-1)
Supplement: Supplementary file 1 — Supplementary materials [file 41398_2018_110_MOESM1_ESM.docx]

**Supplementary materials**

**The results for the rs1421085 polymorphism**

We tested the rs1421085 allele modification of the association between BMI and affective symptoms using the similar SEM framework to the main analysis. We considered rs1421085 C allele a high risk for higher BMI and affective symptoms in an additive genetic model.

The rs1421085 modification model specified in figure 1b had good fit in males (n = 1,236, CFI = .951, RMSEA = .039) and females (n = 1,233, CFI = .942, RMSEA = .043). In both sexes, the rs1421085 C allele was associated with higher BMI at age 11 years, but not with subsequent rates of change (Supplementary figure s2).
