# Supplementary figures and images for: Fat mass and obesity-associated (FTO) rs9939609 polymorphism modifies the relationship between body mass index and affective symptoms through the life course: a prospective birth cohort study
Source: Transl Psychiatry. 2018 Mar 13;8:62. doi: 10.1038/s41398-018-0110-1 (PMC5847566; doi:10.1038/s41398-018-0110-1)

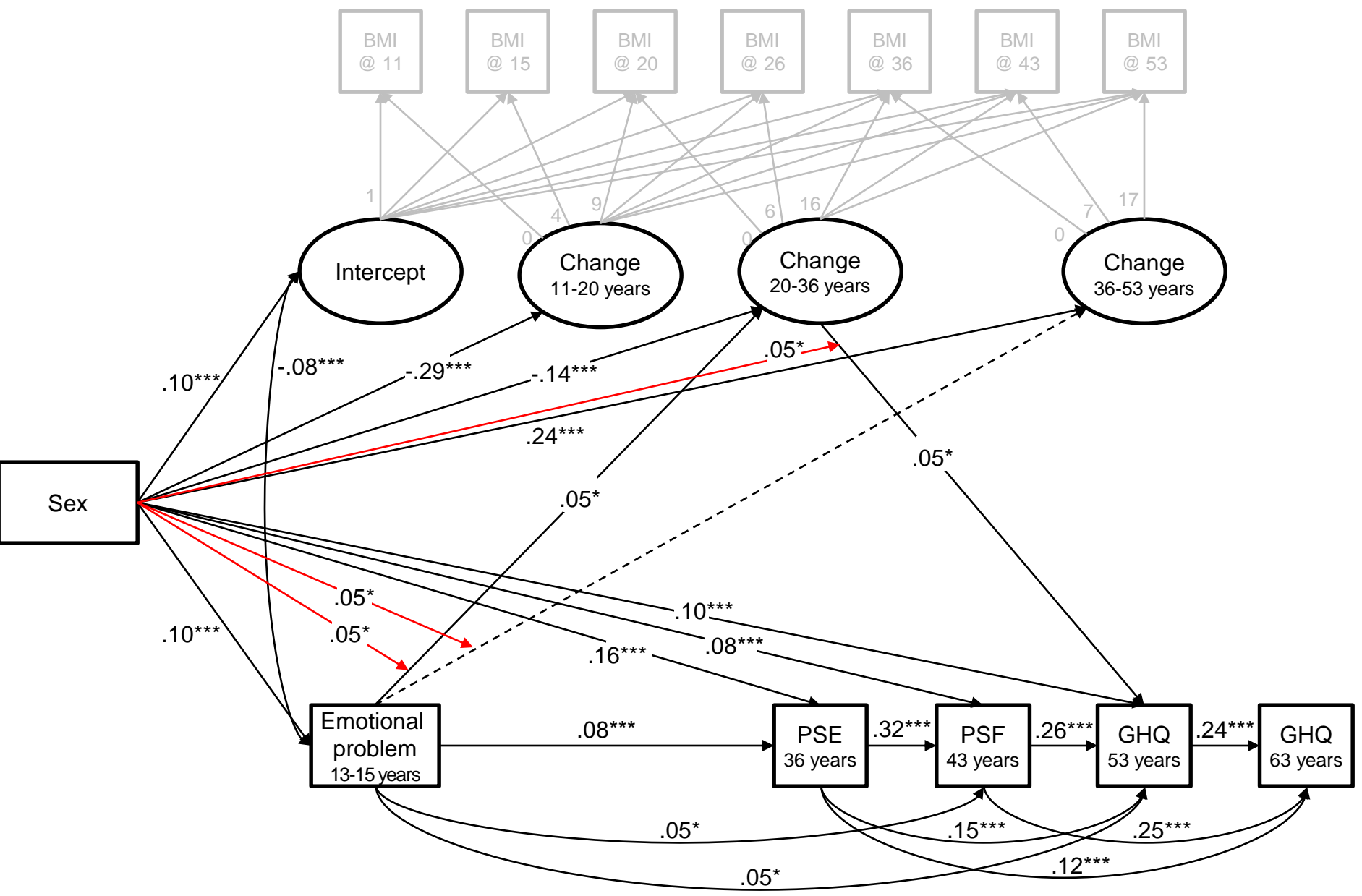

Supplement: Supplementary file 2 — Supplementary figure S1 [file 41398_2018_110_MOESM2_ESM.pdf]

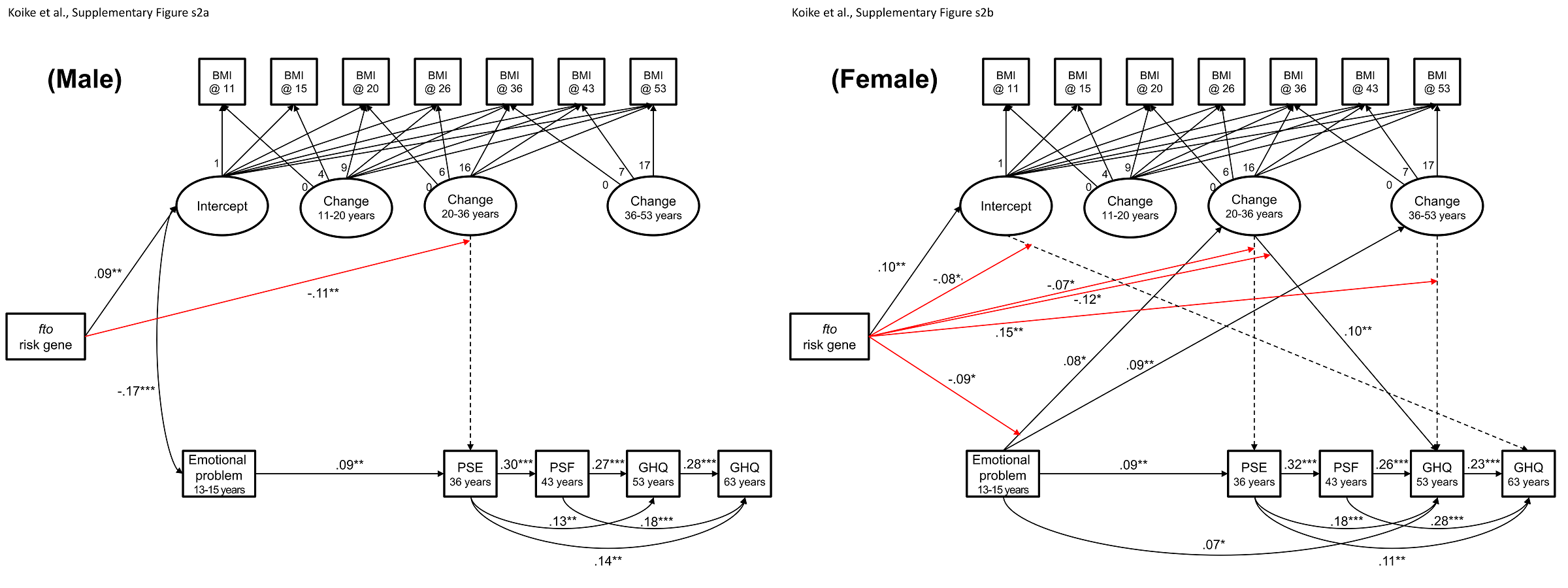

Supplement: Supplementary file 3 — Supplementary figure S2 [file 41398_2018_110_MOESM3_ESM.tif]
