# Supplementary material for: Fat mass and obesity-associated (FTO) rs9939609 polymorphism modifies the relationship between body mass index and affective symptoms through the life course: a prospective birth cohort study
Source: Transl Psychiatry. 2018 Mar 13;8:62. doi: 10.1038/s41398-018-0110-1 (PMC5847566; doi:10.1038/s41398-018-0110-1)
Supplement: Supplementary file 4 — Supplementary Tables [file 41398_2018_110_MOESM4_ESM.docx]

Supplementary Table S1. Association between BMI and affective symptoms and *FTO* rs9939609 genotype in men.

|  |  | Main effect | | | | Interaction by the *FTO* gene | | | |
| --- | --- | --- | --- | --- | --- | --- | --- | --- | --- |
| Dependent variables | Independent variables | Standarized coefficients | SE | Z | P value | Standarized coefficients | SE | Z | P value |
| BMI at age 11 years | FTO risk allele | **0.078** | **0.034** | **2.27** | **0.023** |  |  |  |  |
|  | Emotional problem at 13 and 15 years† | **-0.168** | **0.034** | **-4.95** | **<0.001** |  |  |  |  |
| BMI change between 11―20 years | FTO risk allele | 0.035 | 0.042 | 0.84 | 0.40 |  |  |  |  |
|  | Emotional problem at 13 and 15 years | 0.035 | 0.043 | 0.81 | 0.42 | 0.008 | 0.041 | 0.21 | 0.84 |
| BMI change between 20―36 years | FTO risk allele | -0.012 | 0.036 | -0.32 | 0.75 |  |  |  |  |
|  | Emotional problem at 13 and 15 years | 0.037 | 0.041 | 0.91 | 0.36 | 0.062 | 0.042 | 1.48 | 0.14 |
| BMI change between 36―53 years | FTO risk allele | -0.023 | 0.038 | -0.62 | 0.54 |  |  |  |  |
|  | Emotional problem at 13 and 15 years | 0.002 | 0.044 | 0.04 | 0.97 | 0.017 | 0.042 | 0.41 | 0.69 |
|  | PSE at age 36 years | 0.006 | 0.043 | 0.14 | 0.89 | -0.015 | 0.039 | -0.40 | 0.69 |
|  | PSF at age 43 years | 0.054 | 0.050 | 1.08 | 0.28 | -0.020 | 0.042 | -0.48 | 0.64 |
| Emotional problem at 13 and 15 years | FTO risk allele | 0.013 | 0.031 | 0.43 | 0.67 |  |  |  |  |
| PSE at age 36 years | FTO risk allele | -0.048 | 0.032 | -1.49 | 0.14 |  |  |  |  |
|  | Emotional problem at 13 and 15 years | **0.087** | **0.033** | **2.60** | **0.009** |  |  |  |  |
|  | BMI at age 11 years | 0.062 | 0.033 | 1.86 | 0.063 | 0.011 | 0.039 | 0.27 | 0.79 |
|  | BMI change between 11―20 years | -0.009 | 0.046 | -0.20 | 0.84 | 0.033 | 0.044 | 0.74 | 0.46 |
|  | BMI change between 20―36 years | -0.070 | 0.037 | -1.90 | 0.058 | **-0.095** | **0.035** | **-2.67** | **0.008** |
| PSF at age 43 years | FTO risk allele | 0.003 | 0.031 | 0.11 | 0.91 |  |  |  |  |
|  | Emotional problem at 13 and 15 years | 0.044 | 0.037 | 1.18 | 0.24 |  |  |  |  |
|  | PSE at age 36 years | **0.294** | **0.038** | **7.65** | **<0.001** |  |  |  |  |
|  | BMI at age 11 years | -0.024 | 0.032 | -0.75 | 0.45 | 0.048 | 0.037 | 1.27 | 0.20 |
|  | BMI change between 11―20 years | -0.013 | 0.043 | -0.30 | 0.76 | 0.009 | 0.051 | 0.17 | 0.86 |
|  | BMI change between 20―36 years | 0.007 | 0.042 | 0.18 | 0.86 | 0.018 | 0.038 | 0.46 | 0.64 |
| GHQ at age 53 years | FTO risk allele | -0.053 | 0.028 | -1.90 | 0.057 |  |  |  |  |
|  | Emotional problem at 13 and 15 years | 0.040 | 0.026 | 1.52 | 0.13 |  |  |  |  |
|  | PSE at age 36 years | **0.129** | **0.041** | **3.17** | **0.002** |  |  |  |  |
|  | PSF at age 43 years | **0.267** | **0.037** | **7.17** | **<0.001** |  |  |  |  |
|  | BMI at age 11 years | -0.009 | 0.030 | -0.30 | 0.77 | -0.027 | 0.032 | -0.84 | 0.40 |
|  | BMI change between 11―20 years | 0.024 | 0.036 | 0.67 | 0.50 | -0.025 | 0.034 | -0.73 | 0.47 |
|  | BMI change between 20―36 years | -0.002 | 0.040 | -0.04 | 0.97 | 0.016 | 0.047 | 0.35 | 0.73 |
|  | BMI change between 36―53 years | -0.010 | 0.044 | -0.23 | 0.82 | -0.085 | 0.060 | -1.41 | 0.16 |
| GHQ at age 63 years | FTO risk allele | 0.041 | 0.033 | 1.26 | 0.21 |  |  |  |  |
|  | Emotional problem at 13 and 15 years | 0.042 | 0.034 | 1.23 | 0.22 |  |  |  |  |
|  | PSE at age 36 years | **0.141** | **0.044** | **3.18** | **0.001** |  |  |  |  |
|  | PSF at age 43 years | **0.180** | **0.050** | **3.60** | **<0.001** |  |  |  |  |
|  | GHQ at age 53 years | **0.274** | **0.052** | **5.30** | **<0.001** |  |  |  |  |
|  | BMI at age 11 years | 0.034 | 0.033 | 1.02 | 0.31 | -0.010 | 0.039 | -0.27 | 0.79 |
|  | BMI change between 11―20 years | 0.020 | 0.043 | 0.45 | 0.65 | -0.071 | 0.040 | -1.78 | 0.074 |
|  | BMI change between 20―36 years | 0.075 | 0.042 | 1.79 | 0.074 | 0.063 | 0.041 | 1.54 | 0.12 |
|  | BMI change between 36―53 years | 0.074 | 0.049 | 1.49 | 0.14 | -0.018 | 0.047 | -0.39 | 0.70 |

Bold shows p < .05.

† Correlation between BMI at age 11 years and emotional problem at ages 13 and 15 years.

Abbreviations: BMI, body mass index; PSE, a short version of the Present State Examination; PSF, the Psychiatric Symptom Frequency scale; GHQ, the 28-item version of the General Health Questionnaire; FTO, fat mass and obesity associated gene.

Supplementary Table S2. Association between BMI and affective symptoms and *FTO* rs9939609 genotype in women.

|  |  | Main effect | | | | Interaction by the *FTO* gene | | | |
| --- | --- | --- | --- | --- | --- | --- | --- | --- | --- |
| Dependent variables | Independent variables | Standarized coefficients | SE | Z | P value | Standarized coefficients | SE | Z | P value |
| BMI at age 11 years | FTO risk allele | **0.105** | **0.035** | **2.98** | **0.003** |  |  |  |  |
|  | Emotional problem at 13 and 15 years† | -0.048 | 0.038 | -1.27 | 0.20 |  |  |  |  |
| BMI change between 11―20 years | FTO risk allele | 0.077 | 0.044 | 1.75 | 0.079 |  |  |  |  |
|  | Emotional problem at 13 and 15 years | -0.005 | 0.046 | -0.11 | 0.92 | -0.014 | 0.041 | -0.34 | 0.73 |
| BMI change between 20―36 years | FTO risk allele | -0.056 | 0.036 | -1.55 | 0.12 |  |  |  |  |
|  | Emotional problem at 13 and 15 years | **0.076** | **0.037** | **2.04** | **0.041** | **-0.087** | **0.037** | **-2.37** | **0.018** |
| BMI change between 36―53 years | FTO risk allele | 0.009 | 0.034 | 0.28 | 0.78 |  |  |  |  |
|  | Emotional problem at 13 and 15 years | 0.056 | 0.037 | 1.52 | 0.13 | 0.039 | 0.036 | 1.09 | 0.28 |
|  | PSE at age 36 years | 0.023 | 0.040 | 0.58 | 0.57 | -0.021 | 0.041 | -0.52 | 0.60 |
|  | PSF at age 43 years | 0.051 | 0.038 | 1.35 | 0.18 | -0.001 | 0.038 | -0.02 | 0.99 |
| Emotional problem at 13 and 15 years | FTO risk allele | -0.021 | 0.032 | -0.68 | 0.50 |  |  |  |  |
| PSE at age 36 years | FTO risk allele | -0.023 | 0.030 | -0.76 | 0.45 |  |  |  |  |
|  | Emotional problem at 13 and 15 years | **0.092** | **0.035** | **2.67** | **0.008** |  |  |  |  |
|  | BMI at age 11 years | 0.031 | 0.035 | 0.87 | 0.38 | 0.067 | 0.035 | 1.90 | 0.058 |
|  | BMI change between 11―20 years | 0.017 | 0.043 | 0.39 | 0.70 | 0.061 | 0.039 | 1.59 | 0.11 |
|  | BMI change between 20―36 years | -0.018 | 0.037 | -0.49 | 0.62 | -0.049 | 0.039 | -1.26 | 0.21 |
| PSF at age 43 years | FTO risk allele | -0.046 | 0.027 | -1.67 | 0.095 |  |  |  |  |
|  | Emotional problem at 13 and 15 years | -0.014 | 0.031 | -0.46 | 0.64 |  |  |  |  |
|  | PSE at age 36 years | **0.321** | **0.034** | **9.37** | **<0.001** |  |  |  |  |
|  | BMI at age 11 years | -0.015 | 0.032 | -0.48 | 0.63 | -0.005 | 0.032 | -0.16 | 0.87 |
|  | BMI change between 11―20 years | 0.069 | 0.045 | 1.53 | 0.13 | -0.064 | 0.034 | -1.88 | 0.060 |
|  | BMI change between 20―36 years | 0.010 | 0.033 | 0.31 | 0.76 | **-0.073** | **0.029** | **-2.51** | **0.012** |
| GHQ at age 53 years | FTO risk allele | 0.013 | 0.028 | 0.46 | 0.65 |  |  |  |  |
|  | Emotional problem at 13 and 15 years | **0.070** | **0.029** | **2.44** | **0.015** |  |  |  |  |
|  | PSE at age 36 years | **0.177** | **0.032** | **5.49** | **<0.001** |  |  |  |  |
|  | PSF at age 43 years | **0.261** | **0.035** | **7.44** | **<0.001** |  |  |  |  |
|  | BMI at age 11 years | 0.028 | 0.033 | 0.83 | 0.41 | -0.022 | 0.035 | -0.63 | 0.53 |
|  | BMI change between 11―20 years | 0.034 | 0.042 | 0.80 | 0.42 | 0.037 | 0.038 | 0.97 | 0.33 |
|  | BMI change between 20―36 years | **0.094** | **0.036** | **2.63** | **0.009** | **-0.103** | **0.046** | **-2.23** | **0.025** |
|  | BMI change between 36―53 years | -0.069 | 0.037 | -1.85 | 0.065 | **0.135** | **0.047** | **2.87** | **0.004** |
| GHQ at age 63 years | FTO risk allele | -0.014 | 0.030 | -0.47 | 0.64 |  |  |  |  |
|  | Emotional problem at 13 and 15 years | -0.048 | 0.032 | -1.52 | 0.13 |  |  |  |  |
|  | PSE at age 36 years | **0.111** | **0.036** | **3.10** | **0.002** |  |  |  |  |
|  | PSF at age 43 years | **0.281** | **0.042** | **6.63** | **<0.001** |  |  |  |  |
|  | GHQ at age 53 years | **0.227** | **0.045** | **5.06** | **<0.001** |  |  |  |  |
|  | BMI at age 11 years | 0.056 | 0.039 | 1.44 | 0.15 | **-0.085** | **0.036** | **-2.34** | **0.019** |
|  | BMI change between 11―20 years | -0.048 | 0.043 | -1.12 | 0.26 | -0.012 | 0.034 | -0.36 | 0.72 |
|  | BMI change between 20―36 years | 0.034 | 0.039 | 0.87 | 0.39 | -0.001 | 0.042 | -0.02 | 0.98 |
|  | BMI change between 36―53 years | 0.036 | 0.042 | 0.86 | 0.39 | 0.004 | 0.044 | 0.10 | 0.92 |

Bold shows p < .05.

† Correlation between BMI at age 11 years and emotional problem at ages 13 and 15 years.

Abbreviations: BMI, body mass index; PSE, a short version of the Present State Examination; PSF, the Psychiatric Symptom Frequency scale; GHQ, the 28-item version of the General Health Questionnaire; FTO, fat mass and obesity associated gene.
